# Application of the Inverted Classroom Model for Teaching Pathophysiology to Chinese Undergraduate Medical Students: Usability Study

**DOI:** 10.2196/24358

**Published:** 2021-06-18

**Authors:** Hui Lin, Xiaoping Zeng, Jun Zhu, Zhenzhen Hu, Ying Ying, Yonghong Huang, Hongmei Wang

**Affiliations:** 1 Department of Pathophysiology, School of Basic Medicine Sciences Nanchang University Nanchang China

**Keywords:** pathophysiology, inverted classroom, teaching reform, questionnaire, medical education, undergraduate

## Abstract

**Background:**

The inverted classroom model differs from the traditional teaching model as it reverses the pattern of knowledge transfer and internalization. In recent years, this new teaching model has received much attention in undergraduate medical education. Pathophysiology is a course in the undergraduate Chinese medical curriculum that is critical in bridging basic medical science and clinical medicine.

**Objective:**

The purpose of this study was to investigate the application of inverted classroom in delivering the course on pathophysiology to Chinese undergraduate medical students.

**Methods:**

In the spring semester of 2018, inverted classroom teaching was implemented for second-year clinical medicine students at the College of Medicine at Nanchang University. The topics of hypoxia and respiratory failure were selected for the inverted classroom study. The effect of the inverted classroom on teaching pathophysiology was evaluated using classroom performance metrics, a final examination, and questionnaires.

**Results:**

This study found that students in the inverted classroom group achieved higher scores in their in-course assessments (82.35 [SD 11.45] vs 81.33 [SD 9.51], respectively) and in their final exams (73.41 [SD 10.37] vs 71.13 [SD 11.22], respectively) than those in the traditional lecture-based group, but the scores were not significantly different (*P*=.13, unpaired two-tailed *t* test). There was also no significant difference in the distribution of the score segments in the class quiz (*P*=.09, chi-square test) and in the final exams (*P*=.25, chi-square test) between the 2 groups. Further, most of the students reported that the inverted classroom increased their learning motivation, made them more confident, and helped them understand the content on pathophysiology better. The students in the inverted classroom also improved in their problem-solving skills and teamwork abilities. However, some students from the inverted classroom group also reported that the self-learning and preparatory work before class increased their learning burden.

**Conclusions:**

This study shows the feasibility and promise of inverted classroom for teaching pathophysiology to undergraduate Chinese medical students. The inverted classroom improves students’ learning interests and attitudes toward learning. However, further studies are required to assess the benefits of broader acceptance and implementation of the inverted classroom among Chinese undergraduate medical students.

## Introduction

The inverted classroom is a pedagogical approach in which students study all the necessary class learning content through educational videos and web-based lectures prior to class [1]. In class, the students and an instructor complete the homework questions and participate in collaborative inquiry and interactive communication such as group presentations and discussions. This teaching model subverts the traditional lecture-based instruction with the principle of “teachers in class, homework after class” [2]. The inverted classroom has become the new teaching model, which is widely accepted around the world, including in undergraduate medical education. The application of the inverted classroom has been described in a wide range of disciplines such as medicine, anatomy, nursing, dentistry, and physiology [1,3-7]. The inverted classroom has several key elements that distinguish it from the traditional teaching model [8-10]. First, the teaching concept is inverted from teacher-centered teaching to student-centered learning, where students engage in active self-learning and instructors provide targeted individual guidance [11-13]. Second, the inverted classroom flips the teaching process, wherein students study new knowledge before class. In class, students engage in group collaborative learning and instructors answer questions, which helps students master content knowledge [14]. The inverted classroom also flips the teaching role. Students become independent learners and the instructor provides resources and organizes classroom activities. The instructor is also responsible for providing individualized guidance and answering questions [15]. In addition, the inverted classroom makes full use of web-based teaching resources and databases for online and offline mixed teaching [16].

The Nanchang University Medical College is a middle-level college that recruits hundreds of medical students each year and has many classes. Pathophysiology is an essential basic course in medical education. It involves the study of etiology, pathogenesis, and metabolic and functional changes in disease. Pathophysiology acts as a bridge course connecting basic medicine and clinical medicine courses [17,18]. Pathophysiology teaching outcomes have a nonnegligible effect on the cultivation of medical students’ clinical ability [19]. However, many problems have been encountered during the traditional mode of pathophysiology education, which relies only on didactic lectures and students’ collective listening in our medical college. The traditional mode of teaching pathophysiology does not stimulate students’ interest or their explorative and innovative thinking and there is insufficient interaction between students and teachers. In addition, based on previous experiences, many medical students found that traditional lecture-based pathophysiology courses were boring and the pathophysiology courses were difficult to focus on.

Currently, the number of high-quality web-based teaching resources is increasing in China. Specifically, the Massive Open Online Course of China and the Zhihuishu website offer free courses, and both these websites provide resource support for the application of the inverted classroom [20]. Our teaching team has many years of experience in teaching with multimedia and has launched many web-based teaching courses. Moreover, students’ learning abilities are improving and their learning styles are becoming increasingly diverse. These factors have laid a solid foundation for the implementation of the inverted classroom for pathophysiology education. Thus, the inverted classroom as a new teaching model could be an effective strategy for teaching pathophysiology. To test this possibility, we explored the feasibility and effectiveness of the inverted classroom for teaching pathophysiology.

## Methods

### Subjects and Ethical Approval

This study was conducted at the College of Medicine at Nanchang University, China; 207 second-year (2017/2018 academic year) students majoring in clinical medicine registered for the pathophysiology course and participated in this study. In their first year, students completed pathology, human anatomy, histology-embryology, medical biology, biochemistry, medical microbiology, parasitology, immunology, genetics, and physiology courses. The final examination scores of the students in these courses were not significantly different. During this study, students studied pathoanatomy, pathophysiology, and pharmacology and had not participated in inverted classrooms before. Students were randomly divided into an inverted classroom group (n=100) and a control group (traditional lecture-based classroom, n=107). The age of the students ranged from 19 to 21 years. There was no difference in the admission scores between the 2 groups. The students’ learning levels and abilities were nearly equal. All students submitted their informed consent before this study, and this study was approved by the Committee of Nanchang University (NCUJGLX-16-86). Completion of the survey was considered implied consent of participation and students’ participation in the survey was optional.

### Curriculum Description and Study Design

The eighth edition of Pathophysiology published by People’s Medical Publishing House and edited by Wang Jianzhi and Qian Ruizhe was used for this course [21]. Two sections of the textbook were selected to implement an inverted classroom for this study, namely, hypoxia and respiratory failure. These topics were selected based on feedback from a previous study and the student questionnaire. The hypoxia section consisted of 3 lectures, which covered the classification, etiology, and mechanisms of hypoxia; metabolic and functional alterations in the body; and the prevention and treatment of hypoxia. The respiratory failure section consisted of 4 lectures, which covered the classification, etiology, and pathogenesis of respiratory failure; metabolic and functional alterations in the body; and the prevention and treatment of respiratory failure. Hypoxia and respiratory failure are associated with each other, thereby making these topics easy for students to prepare and implement in an inverted classroom.

**Figure 1:**
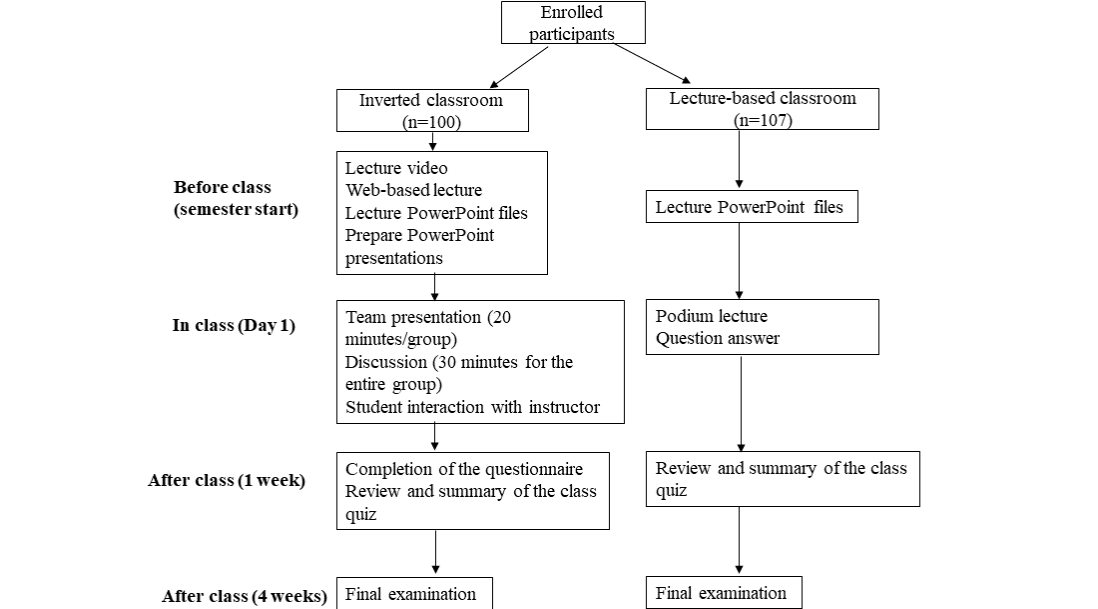
Flow diagram illustrating the inverted classroom and traditional lecture-based classroom models.

In the inverted classroom group (Figure 1), the students were divided into small teams with 7 students per team. For the preclass student preparation, the instructors provided study materials such as web-based lectures (videos), PowerPoint lectures, teaching requirements and objectives, knowledge points, and the materials from the chapters according to the syllabus requirements. The web-based videos included hypoxia and respiratory failure sections, which had been recorded by the faculty in the department and were provided through the Massive Open Online Course of China. Each team had to watch the lecture and prepare a PowerPoint presentation (20 minutes) for in-class discussion. The assignment was posted 1 week before the class. The class started with a brief review and an outline of the lecture by the instructor. Each team made a presentation to introduce the lecture concepts and pose questions. After all the teams completed their presentations, each team answered questions and discussed them for 30 minutes. The instructor also joined the discussion and summarized the concepts at the end. For the traditional lecture-based classroom (Figure 1), the instructor gave a podium-style lecture about hypoxia and respiratory failure. Additionally, the students in this group were also encouraged to watch the web-based lectures and preview the 2 sections.

### Statistical Analysis

After the class, the knowledge of the students in both the groups was tested on hypoxia and respiratory failure with a quiz to evaluate their performance. A web-based questionnaire using Questionnaire Star was used to collect data on students’ feedback about the inverted classroom. In addition, the students’ performance was compared based on their final exam scores. All statistical analyses were performed using GraphPad Prism 9 statistical software. Students’ scores in the quiz and final examination were compared between inverted classrooms and traditional classrooms by using the unpaired two-tailed *t* test. The distribution of the score segments (<60, 60-69, 70-79, 80-89, 90-100) of the 2 groups was determined using the chi-square test. Results were considered significant at *P*<.05.

## Results

### Quiz Findings

**Figure 2:**
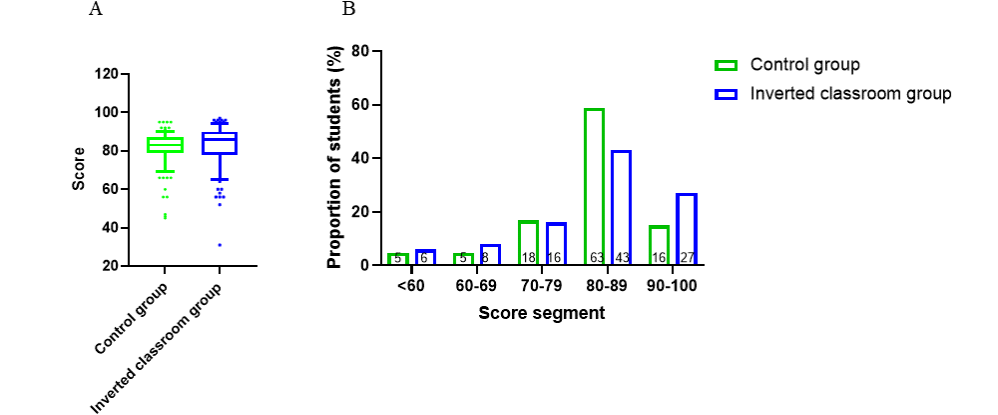
A. Comparison of students’ test scores in class quiz in the control and inverted classroom groups. An unpaired two-tailed *t* test was used to compare the differences between the 2 groups (*P*=.49). B. Students' grades in the class quiz divided into 5 segments with the proportion of students in each segment.

**Figure 3:**
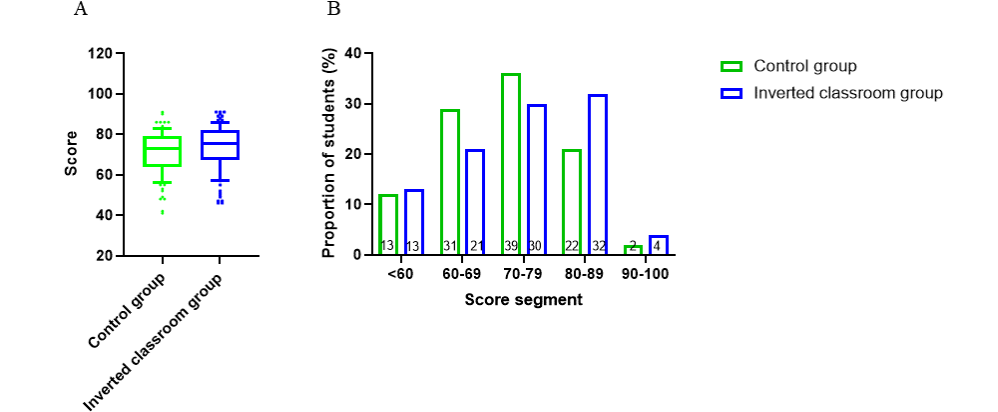
A. Comparison of students’ test scores in the final exam in the control and inverted classroom groups. An unpaired two-tailed *t* test was used to compare the differences between the 2 groups (*P*=.13). B. Students' grades in the final exam divided into 5 segments with the proportion of students in each segment.

To evaluate the students’ performance, a quiz on hypoxia and respiratory failure was administered immediately after the class. The students in the inverted classroom group received higher scores (82.35 [SD 11.45]) than those in the traditional lecture-based group (81.33 [SD 9.51]), but the difference in the scores was not statistically significant (unpaired two-tailed *t* test, *P*=.50, Figure 2A). Scores were divided into 5 score segments as outlined in Figure 2B. The score distributions in the 2 groups were compared and no statistically significant difference was found in the score segment distribution (*P*=.09, inverted classroom group vs control group, chi-square test). The proportions of students in the inverted class in the 5 performance segments were as follows: 6.0% (6/100, score <60), 8.0% (8/100, score=60-69), 16.0% (16/100, score=70-79), 43.0% (43/100, score=80-89), and 27.0% (27/100, score=90-100). The proportions of the students in the control class in the 5 performance segments were as follows: 4.7% (5/107, score<60), 4.7% (5/107, score 60-69), 16.8% (18/107, score 70-79), 58.9% (63/107, score 80-89), and 14.9% (16/107, score 90-100). The data showed that the proportion of students in the inverted class with scores between 90 and 100 was 12.1% higher than that in the control class. However, the proportion of students in the inverted class with scores between 80 and 89 was 15.6% lower than that in the control class. Then, the final examination scores between the 2 groups were compared. The results showed that the average score of the students in the inverted classroom group (73.41 [SD 10.37]) was higher than that of the students in the traditional lecture-based group (71.13 [SD 11.22], unpaired two-tailed *t* test, *P*=.13, Figure 3A), but this difference was not statistically significant. Moreover, the scores of the students were divided into 5 score segments (Figure 3B) and the distribution was not found to be statistically different (*P*=.25, inverted classroom group vs control group, chi-square test). However, we found that the proportion of students in the inverted class with scores between 80-89 and 90-100 was 11% and 2% higher than that in the control class, respectively. This finding suggests that students in the inverted classroom group received higher scores than those in the control class group.

### Questionnaire Analysis

To evaluate the students’ attitudes toward and the perspectives of the inverted classroom mode, a web-based survey was administered to the inverted classroom group at the end, and 98 responses were collected. The survey results showed that most students believed that the inverted classroom model increased their opportunities for interactions with their classmates and teachers (Table 1); however, some students noted that a few students did not actively participate in team learning and discussions. Moreover, the survey results showed that the inverted classroom stimulated students’ interests and enabled them to have a better grasp of the course content. The inverted classroom improved the students’ self-learning and problem-solving abilities. In addition, the inverted classroom improved the quality of teaching and study. Finally, the students also proposed the following constructive suggestions: (1) considering the limited time of students, the instructor should coordinate with instructors of other subjects before conducting the inverted classroom; (2) students should be provided more preparation time; (3) the time for group presentations and discussions immediately after the presentations should be extended, (4) the instructor should resummarize the course content so that the students can better the master course content; and (5) social media such as WeChat and QQ groups should be used to discuss questions during the study, which would also improve interactions with classmates and instructors.

**Table 1:** Perceptions of the medical students toward the inverted classroom model on selected topics (n=98).

Questions, responses			Values
**The traditional teacher-led classroom style guarantees teaching efficiency.**			
	Agree	49	
	Neutral	41	
	Disagree	5	
	Hard to say	3	
**The learning style of inverted classroom is very helpful for studying the topics.**			
	Agree	29	
	Neutral	43	
	Disagree	26	
**The inverted classroom has a positive or negative impact on you.**			
	Positive, active, and more effective	59	
	Negative, unable to concentrate	39	
**The inverted classroom has improved self-active study.**			
	Yes	76	
	No	22	
**Can inverted classroom increase your motivation for learning?**			
	Agree	62	
	Disagree	36	
**Does the self-study before class in the inverted classroom increase your learning burden?**			
	Yes	39	
	No	59	
**In class, can your questions be solved?**			
	Yes	12	
	Some are resolved	84	
	No, not at all	2	
**The reason for being unable to complete the assignment.**			
	Not enough time	52	
	Do not know	52	
	Do in class	35	
	Other	10	
**In self-study, what do you think is most helpful to you?**			
	Textbook analysis	75	
	Video lecture	48	
	Portal learning	34	
	Others	5	
**Can you use the time after class to complete the self-study goals assignment?**			
	Yes	33	
	Some	35	
	Finish after required	30	
**How do you think the inverted classroom contributes to your learning aid?**			
	Self-learning after class	52	
	Problem-solving in class	46	

## Discussion

### Principal Findings

The student-centered inverted classroom has been widely used in medical education and is one of the teaching reform models currently being implemented in college [22-24]. In an inverted classroom, students are encouraged to spend their spare time learning and improving their learning efficiency. The inverted classroom also allows students to seek answers based on questions raised during their studies [25]. Moreover, students can develop individual learning plans according to their unique situations, which aids learning efficiency and better academic performance [26,27]. To provide a new teaching model for the pathophysiology course and to promote the development of pathophysiology education, we designed a relatively complete teaching scheme based on the inverted classroom model for the hypoxia and respiratory failure sections of the pathophysiology course. By implementing the inverted classroom, classroom quiz, questionnaire, and final exam, we found that students’ performance increased in terms of their abilities and interests, their best efforts, and their presentation of content in the classroom. Collectively, the inverted classroom for pathophysiology education not only strengthened mutual assistance and solidarity among students but also enhanced the interactions between teachers and students.

In the implementation process, we also identified some issues: (1) in group discussion and preparations, the group leaders performed most of the work rather than each student contributing to the team work, (2) students only studied the materials provided by the teacher and did not search for additional supplementary materials, (3) some students with less active learning styles did not study sufficiently before class and did not perform well in the classroom, and (4) some students complained that there were too many courses undergoing teaching reforms; therefore, it took a substantial amount of time to prepare for their classes, which increased their learning burden. Therefore, the student-centered inverted classroom should establish new requirements for student learning, including student learning initiatives and the rational use of learning resources such as media, internet, and electronic books. In this way, students can obtain more knowledge and stimulate their interest and motivation. In inverted classrooms, the student’s self-learning ability is strengthened and the knowledge is more effectively retained, as shown by the high scores in the class quiz and final exams. In inverted classrooms, students must pay attention in class as they are actively participating in the presentations and discussions. In contrast, in traditional classrooms, the instructor gives lectures and students are prone to inattention and distraction, with less time for discussion.

The application of the inverted classroom teaching model also introduces additional requirements for instructors. Pathophysiology is an important bridge between basic medicine and clinical medicine. Instructors should be familiar with the entire curriculum system, apply a holistic approach, connect knowledge points in tandem, and guide students in learning the content of each chapter. For instance, hypoxemia occurs in respiratory failure, which also leads to acid-base balance disorders. In addition, instructors should pay attention to the links between various disciplines. To study pathophysiology, students should have knowledge of the normal human body, functions, and metabolism from the point of view of physiology and biochemistry.

### Strengths of This Study

The inverted classroom breaks the traditional classroom “teaching-learning” model and effectively compensates for some of the shortcomings of the undergraduate teaching model. This teaching model can improve the quality of pathophysiology education, fulfill the needs of students, and bring medical classroom learning closer to clinical practice. This teaching model inspires students’ innovative thinking and cultivates medical talent with high learning ability. The development of an inverted classroom in pathophysiology is conducive to improving a student’s self-study ability. Students master not only the knowledge but also the methods of obtaining knowledge, which can better bridge the transition from basic medicine to clinical medicine.

### Limitations of This Study

This study had the following limitations. First, the number of students in this study was relatively small. We could have obtained more convincing conclusions from this study had an inverted classroom been conducted with more students. Second, since other courses (apart from pathophysiology) were also taught using the inverted classroom model, the burden of the students increased during this study. In addition, students had only 4 weeks to review and then take the final exam after the course ended. Other factors such as independent study may have also affected the results. Third, we selected only 2 topics in the pathophysiology course for the inverted classroom.

### Conclusion

We are still in the preliminary stage of applying student-centered inverted classrooms in teaching pathophysiology. The extensive implementation of inverted classrooms requires further research and exploration. The inverted classroom can provide a new teaching model for pathophysiology and other clinical medicine majors and promote the quality of teaching and the development of curriculums in pathophysiology.
